# Genetic alteration and clonal evolution of primary glioblastoma into secondary gliosarcoma

**DOI:** 10.1111/cns.13740

**Published:** 2021-10-04

**Authors:** Jie Li, Yu‐Hang Zhao, Su‐Fang Tian, Cheng‐Shi Xu, Yu‐Xiang Cai, Kai Li, Yan‐Bing Cheng, Ze‐Fen Wang, Zhi‐Qiang Li

**Affiliations:** ^1^ Brain Glioma Center Zhongnan Hospital of Wuhan University Wuhan China; ^2^ Department of Physiology Wuhan University School of Basic Medical Sciences Wuhan China; ^3^ Department of Pathology Zhongnan Hospital of Wuhan University Wuhan China; ^4^ Wuhan Frasergen Bioinformatics Company Limited Wuhan China

**Keywords:** clonal evolution, mutation signature, primary glioblastoma, radiotherapy, secondary gliosarcoma

## Abstract

**Aims:**

Secondary gliosarcoma (SGS) rarely arises post treatment of primary glioblastoma multiforme (GBM), and contains gliomatous and sarcomatous components. The origin and clonal evolution of SGS sarcomatous components remain uncharacterized. Therapeutic radiation is mutagenic and can induce sarcomas in patients with other tumor phenotypes, but possible causal relationships between radiotherapy and induction of SGS sarcomatous components remain unexplored. Herein, we investigated the clonal origin of SGS in a patient with primary GBM progressing into SGS post‐radiochemotherapy.

**Methods:**

Somatic mutation profile in GBM and SGS was examined using whole‐genome sequencing and deep‐whole‐exome sequencing. Mutation signatures were characterized to investigate relationships between radiochemotherapy and SGS pathogenesis.

**Results:**

A mutation cluster containing two founding mutations in tumor‐suppressor genes *NF1* (variant allele frequency [VAF]: 50.0% in GBM and 51.1% in SGS) and *TP53* (VAF: 26.7% in GBM and 50.8% in SGS) was shared in GBM and SGS. SGS exhibited an overpresented C>A (G>T) transversion (oxidative DNA damage signature) but no signature 11 mutations (alkylating‐agents – exposure signature). Since radiation induces DNA lesions by generating reactive oxygen species, the mutations observed in this case of SGS were likely the result of radiotherapy rather than chemotherapy.

**Conclusions:**

Secondary gliosarcoma components likely have a monoclonal origin, and the clone possessing mutations in *NF1* and *TP53* was likely the founding clone in this case of SGS.

## Graphical abstract

This study presents the first comprehensive genomic profile and clonal architecture of tumor evolution from glioblastoma multiforme to Secondary gliosarcoma (SGS). The data support not only the monoclonal origin of gliomatous and sarcomatous components in SGS but also the causal relationship between therapeutic radiation and SGS pathogenesis.

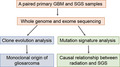

## 1 INTRODUCTION

Gliosarcoma, a rare variant of glioblastoma multiforme (GBM) containing both gliomatous and sarcomatous components, is considered a Grade IV neoplasm according to the WHO classification.^1^ Gliosarcoma is clinically similar to GBM and affects mainly 50–70‐year‐old adults, with a higher proportion found in men.^2^ Patients with gliosarcoma and GBM show similarly poor survival outcomes and are typically treated using the same regimen including maximal safe resection, and concomitant radiotherapy and chemotherapy.^3^, ^4^, ^5^, ^6^

The pathogenesis of gliosarcoma remains unclear. One hypothesis states that the gliomatous and sarcomatous components originate from different progenitor cells, with the sarcomatous element arising from vascular smooth muscle cells, pluripotent mesenchymal cells of the perivascular adventitia, fibroblasts, or even histolytic cells.^7^, ^8^, ^9^, ^10^, ^11^ This concept of biclonal origin of gliosarcoma is based mainly on distinct histological and morphological characteristics of two components. Another prevalent hypothesis, which states that both components share a monoclonal origin from a common progenitor cell, is supported by several studies showing identical genetic alterations (including mutations in *TP53* or *PTEN*, and copy number variation (CNV) of *P16* or *CDK4*) in the two components.^12^, ^13^, ^14^, ^15^ Several other genetic variations, which are common in GBM, have also been found in both gliomatous and sarcomatous components of SGS, including CNVs on chromosomes 7, 9p, 9q, 13q, 20q, and X.^13^, ^16^

Most gliosarcomas are de novo (primary), while those arising after chemoradiation of primary GBM are termed secondary gliosarcomas (SGS). Radiation therapy is conventionally used for the treatment of GBM. Various radiation‐induced intracranial tumors include meningiomas, gliomas, fibrosarcomas, and gliosarcomas.^17^ Radiation‐induced tumors arise within or adjacent to the previously irradiated field and exhibit a histologically distinct type from the original tumor.^18^ SGS tumors occurring after treatment of GBM are distinguishable from radiation‐induced gliosarcomas, which develop after intracranial radiation without prior history of GBM. A clinical study by Han et al.^19^ showed that patients with SGS and radiation‐induced gliosarcomas show distinct survival outcomes and latency periods between radiation and diagnosis of gliosarcoma. In that study, all the patients with SGS had undergone radiotherapy, suggesting that radiation may act as a critical agent for the induction of gliosarcoma. Deb et al. found that *TP53* mutations were present in both gliomatous and sarcomatous components of SGS,^20^ supporting the theory for a monoclonal SGS origin. However, the association between anti‐GBM radiotherapy and histogenesis of SGS sarcomatous elements remains unclear.

Although the clinicopathological, molecular, and genetic characteristics of GBM and SGS have been described previously,^21^, ^22^ few studies have examined the genomic alteration and clonal evolution involved in the progression of original GBM into SGS. In this study, we describe a patient with primary GBM that progressed to SGS 10 months after radiochemotherapy. To elucidate the clonal origin of SGS, we performed whole‐genome sequencing (WGS) and ultra‐deep whole‐exome sequencing (WES) using the paired primary GBM and SGS specimens obtained from this patient. Comparison of primary GBM profile against that of SGS using genomic analysis indicated that this case of SGS possessed a monoclonal origin. Mutation signature analysis indicated that therapeutic radiation significantly contributed to the genesis of SGS examined in our present study. To the best of our knowledge, this study is the first to describe a comprehensive genomic profile and clonal architecture of tumor evolution from GMB to SGS.

## 2 MATERIALS AND METHODS

### 2.1 Patient and samples of tumor tissue

**FIGURE 1:**
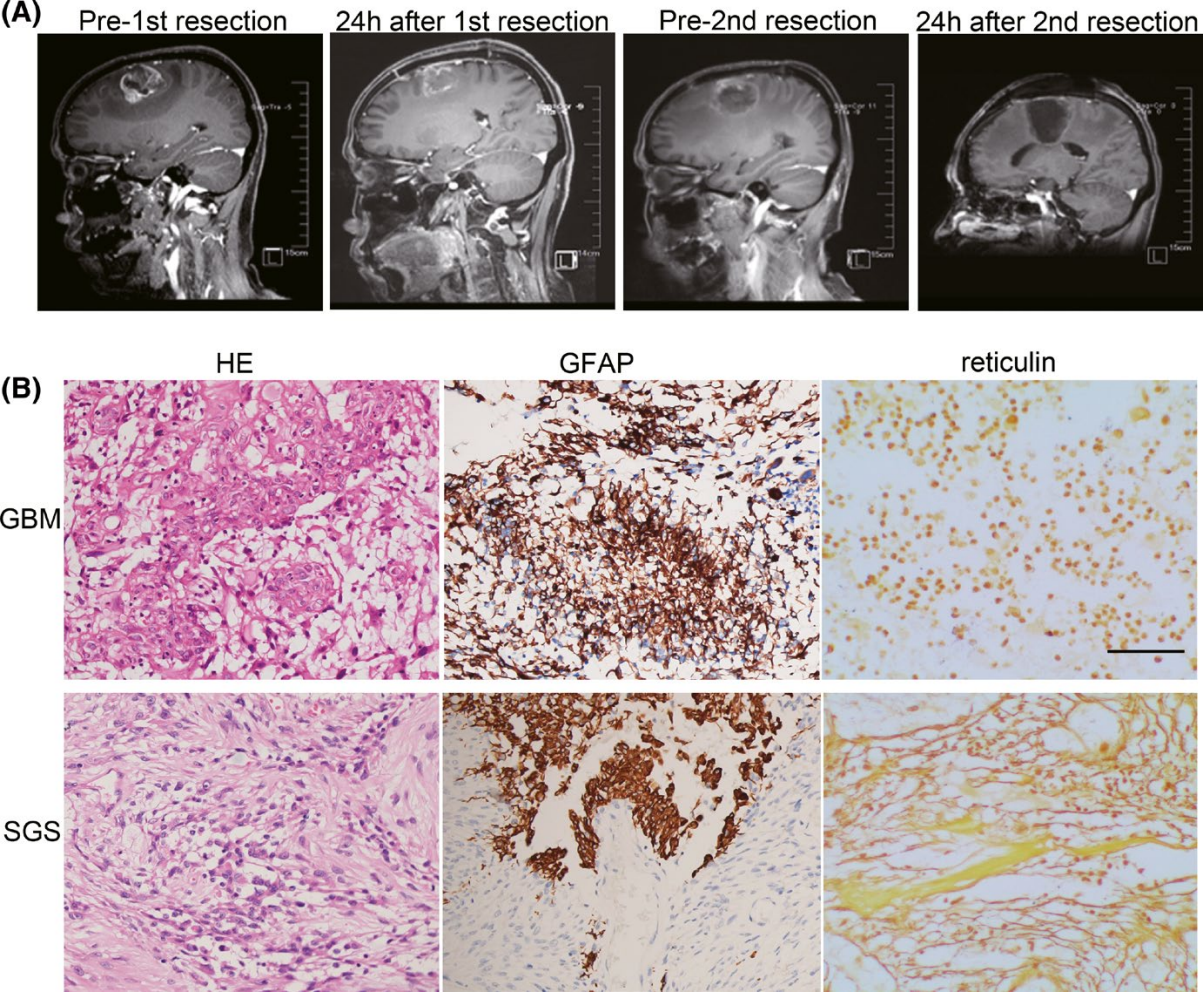
Magnetic resonance imaging and histopathological staining of brain‐tumor tissues. (A) Sagittal T1‐weighted contrast‐enhanced MRI imaging of initial and recurrent tumors. (B) Representative images of primary glioblastoma (GBM) and secondary gliosarcoma (SGS) tissues stained using hematoxylin and eosin (HE), and immunolabeled using antibodies to glial fibrillary acidic protein (GFAP) and reticulin

This study was approved by the Ethics Committee of Zhongnan Hospital of Wuhan University (No.2019048) and conducted in accordance with the ethical principles of the Declaration of Helsinki. The patient provided her signed written informed consent for the use of tumor tissues for research purposes. A 59‐year‐old woman was admitted to the Department of Neurosurgery, Zhongnan Hospital, Wuhan University, in July 2016. The chief complaint was weakness in the left arm and leg that persisted for 14 days. Physical examination showed that the muscle strength of the left upper and lower extremities was grade 4. Preoperative MRI scan showed a contrast‐enhanced lesion with obvious edema in the right frontal and parietal lobes (Figure 1A). Awake craniotomy combined with neuro‐navigation and fluorescent staining was performed in July 2016. Concurrent radio‐chemotherapy (PTV‐GTV 60 Gy/30 F, PTV‐CTV 54 Gy/30 F, temozolomide 75 mg/m^2^/day) was started at 3 weeks post‐resection and continued for the duration of 6 weeks, followed by adjuvant chemotherapy using 200 mg/m^2^ temozolomide for four cycles. Regular follow‐up MRI, defined as a brain MRI performed at 3 months post‐resection, showed a localized lesion at the same location of original tumor in December 2016 (Figure 1A). The patient was then administered Bevacizumab (10 mg/kg) plus dose‐dense temozolomide (50 mg/m^2^/day) for two cycles. However, the muscle strength of the left arm and leg had decreased again in April 2017, and then a second surgery was performed.

### 2.2 Next‐generation sequencing

Genomic DNA was extracted using DNAzol (GibcoBRL) from frozen tumor tissue and peripheral blood mononuclear cells obtained from this patient. The quality of the obtained DNA was analyzed using a NanoDrop 2000 Spectrophotometer and Qubit Flex Fluorometer (Thermo Fisher Scientific). For WES, all exons in protein‐coding regions and flanking intron regions were captured and enriched using a SureSelect Human V6 Exome Capture Kit (Agilent). Library construction and high‐throughput sequencing were conducted on an Illumina NovaSeq 6000 sequencing platform per manufacturer's protocol.

### 2.3 Mutation detection

Low‐quality reads and sequencing adaptors were removed from raw sequencing data to obtain clean reads. Human reference genome GRCH37, downloaded from the Ensembl database, was used as reference to align reads using Burrows‐Wheeler Aligners (BWA).^23^ The Picard tools were used to sort the alignment and remove duplicate reads from the aligned results. GATK‐Mutect2 was employed to detect somatic single nucleotide variants (SNVs) and to call small insertions and deletions (indels).^24^ SNVs and indels were annotated by variant effect predictor (VEP) and ANNOVAR.^25^, ^26^

### 2.4 Detection of structural variants

Structural variants (SVs) were predicted by DELLY2 using BAM files containing alignments of paired tumor and peripheral‐blood control samples.^27^ Tumor‐specific somatic SVs were identified by comparing SVs found in samples of GBM/SGS tissues against those in control blood samples. All somatic SVs were annotated using ANNOVAR to determine their type and functional classification.

### 2.5 Detection of copy number variants

Somatic copy number variants (CNVs) were detected by Control‐FREEC using aligned reads of matched tumor and peripheral‐blood control samples as input. Control‐FREEC can analyze overdiploid tumor samples and samples contaminated by normal cells.^28^ Control‐FREEC was also used to identify loss‐of‐heterozygosity events. All detected CNVs were consequently annotated according to the Decipher, DGV, and ISCA databases.

### 2.6 Mutational signature analysis

Mutational signature analysis was performed using MuSiCa to visualize the somatic mutational profile and extract the contribution of reported mutational signatures.^26^ All SNVs identified using WES deep‐sequencing data were utilized as input for analysis using MuSica.

### 2.7 Clonal evolution analysis

Deep‐sequenced mutations in WES data were used for the analysis of clonal evolution. Somatic non‐silent SNVs (nonsynonymous mutations, nonsense mutations, and mutations affecting splicing sites) with VAF > 0.1% in the samples of GBM or SGS tissues were selected for the inference of clonal population structures. Pyclone was used to identify and quantify clonal populations in our samples of GBM and SGS tissues.^26^

### 2.8 Analysis of mutation incidence in GBM patients from databases

To determine whether the mutations identified in this study commonly occur in primary GBM, we screened the data of mutations in several available databases including the Chinese Glioma Genome Atlas (CGGA, http://www.cgga.org.cn), The Cancer Genome Atlas (TCGA, http://cancergenome.nih.gov), and GSE16011 from Gene Expression Omnibus (GEO, https://www.ncbi.nlm.nih.gov/geo/). These databases provide the molecular, genomic, and clinical data of the patients with different types of tumors, and are commonly used to screen the biomarkers with implications of prognosis and/or therapy resistance.^29^, ^30^, ^31^

## 3 RESULTS

### 3.1 Molecular characteristics of glioblastoma multiforme and secondary gliosarcoma

The patient described in our present study was diagnosed with primary GBM using standard and molecular pathological analyses. PTEN (T232fs) and P53 (V203M) mutations and EGFR amplification were identified in the primary tumor tissue, but mutations in IDH1/2, BRAF, and TERT, and 1p/19q co‐deletion were not detected. MGMT promoter methylation status was negative as indicated by pyrosequencing. The patient recovered, showing a Karnofsky Performance Scale score of 90 after the first gross total resection, but was diagnosed with SGS 10 months after the first GBM resection, and died from tumor progression 5 months after she was diagnosed with gliosarcoma. The recurrent tumor contained GFAP‐expressing gliomatous tissue and reticulin‐rich sarcomatous elements (Figure 1B). The molecular and genetic characteristics of SGS tissue were identical to those of primary GBM. Many literatures report that the genetic profile of gliosarcoma is similar to that of GBM, except for absent or minimal EGFR amplification and rare cases of IDH mutations.^14^, ^32^ In this patient, EGFR amplification was identified in both primary GBM and SGS using fluorescence in situ hybridization (FISH).

### 3.2 Genomic analysis

To understand the genomic‐alteration and clonal‐evolution processes involved in the progression of primary GBM into SGS, we used WGS to analyze the paired samples of primary GBM and recurrent SGS, and samples of peripheral blood mononuclear cells used as matching controls. WGS has a sequencing depth of approximately 30×. To increase the quantification accuracy of variant‐allele frequency and detection sensitivity for low‐abundance variants, we used ultra‐deep WES to generate more than 50 Gbp of sequencing data for both GBM and SGS. Alignment of the WES data obtained in this study covered 95% of the exome region with >100× depth; average sequencing depth for GBM and SGS was 553× and 652×, respectively.

A total of 3729 somatic mutations were identified in the GBM WES data, of which 353 were non‐silent SNVs (missense, nonsense, and splicing‐site changing) and 80 were indels in the coding regions (Data S1). In the SGS WES data, we identified 1050 mutations, including 186 non‐silent SNVs and 24 coding indels (Data S1). Among the non‐silent mutations, 42 were shared between the two samples, while 391 and 168 were specific to GBM or SGS, respectively. Tumor mutation burden, calculated as the number of mutations per megabase, was 7.2 for GBM and 3.5 for SGS.

**FIGURE 2:**
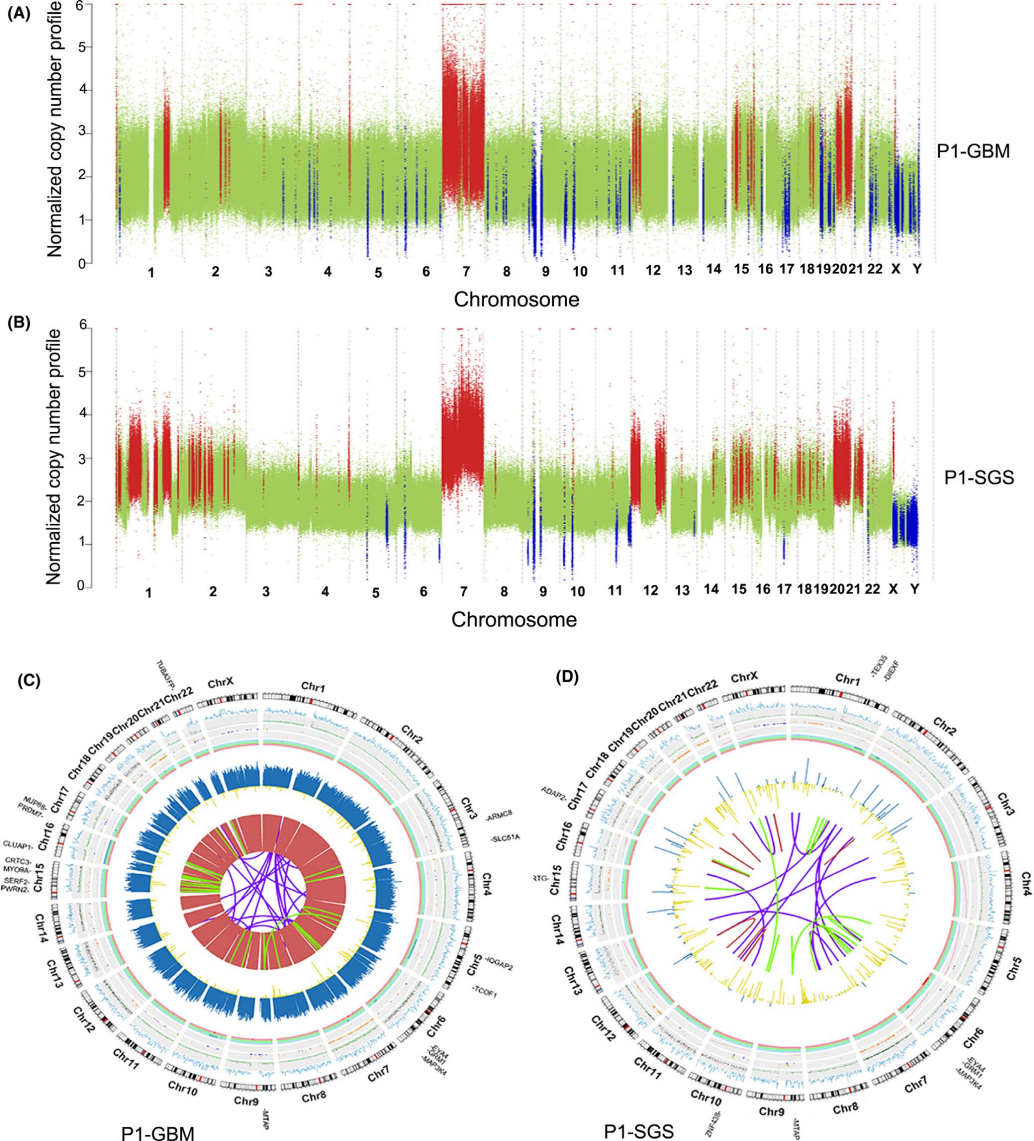
Visualization of normalized‐copy‐number profile and whole‐genome somatic single nucleotide variants (SNVs), indels, copy number variations (CNVs), and structural variations (SVs) in glioblastoma multiforme (GBM) and secondary gliosarcoma (SGS) tissues. (A, B) Relative‐copy‐number statuses across each chromosome are indicated by colored dots in GBM (A) and SGS tissue samples (B). The red dots represent a region with copy number gain. The blue dots represent a region with copy number loss. The green dots represent a region without any copy number alterations. (C, D) Circos plot illustrating relationships between amplification status, translocations, and candidate driver genes in GBM (C) and SGS (D). From outside to inside: 1. Translocation, inversions, and gene fusions; 2. small insertions and deletions; 3. insertions; 4. duplications; 5. deletions; 6. loss of heterozygosity; 7. copy number variations; 8. sequencing depth; 9. SNV density; 10. chromosome names; 11. driver genes

Whole‐genome sequencing data were used to identify whole‐genome SNVs, small indels, and larger variants including CNVs and SVs. We identified a total of 20,191 somatic mutations (18,240 SNVs and 1951 indels) in GBM and 12,240 somatic mutations (11,078 SNVs and 1162 indels) in SGS (Data S2). Analysis of CNVs revealed 82 copy‐number losses (seven focal deletions and 75 broad deletions) and 318 copy‐number gains (159 focal amplification and 159 broad amplification) in GBM. Copy‐number loss and gain events in SGS were 50 (nine focal Del and 41 broad Del) and 542 (220 focal AMP and 322 broad AMP), respectively. Whole‐genome copy number (CN) and CNVs were plotted and are shown in Figure 2A,B. Notably, extensive copy number amplifications were observed on chromosome 7 in both GBM and SGS. Other shared CNVs between GBM and SGS were a CN gain on chromosomes 1, 12, 15, and 20, and CN loss on chromosomes 5, 6, 9–11, and X. GBM specific‐CNVs were mainly CN losses on chromosomes 8, 17, and 19 (Figure 2A), while SGS‐specific CNVs involved CN gains on chromosomes 1, 2, 12, 16, 18, and 20 (Figure 2B). SVs, including large‐fragment insertions, deletions, duplications, inversions, and translocations, were also detected in WGS data. The somatic variants of all types were visualized using Circos plots and are shown in Figure 2C,D.

### 3.3 Clonal evolution

**FIGURE 3:**
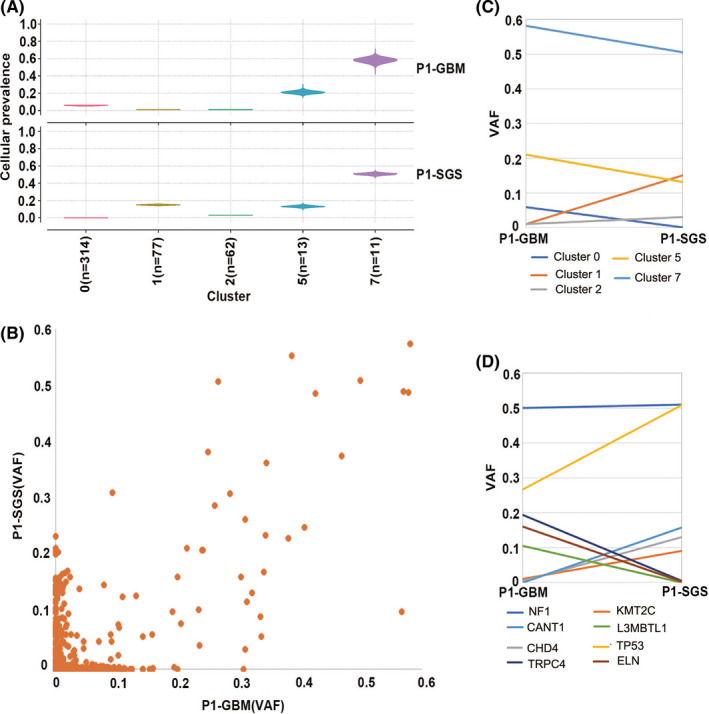
Clustering and clonal evolution of somatic mutations. (A) Clustering of somatic single nucleotide variants (SNVs) in glioblastoma multiforme (GBM) and secondary gliosarcoma (SGS). Vertical axis indicates the inferred cellular prevalence of each cluster. (B) Scatter plot of variant allele frequencies (VAFs) for somatic mutations. Horizontal axis indicates SNVs with VAFs in GBM, and vertical axis indicates SNVs with VAFs in SGS. (C) Change in VAFs inferred for each cluster, from GBM to SGS. (D) Changes in VAFs of several driver genes in GBM and SGS

Ultra‐deep WES allowed us to accurately quantify variant allele frequency (VAF) in order to estimate the size of tumor clonal population in GBM and SGS. Somatic non‐silent SNVs, identified in GBM or SGS, were used for the analysis of tumor clonal evolution (Data S3). Based on the clustering of mutation allele frequency, we inferred five clones (clusters 0, 1, 2, 5, and 7) having a clonal size larger than 10, each containing different sets of mutations (Figure 3A, and Data S3). Then, VAFs of the SNVs in these clusters were plotted to show the prevalence of mutations during tumor progression (Figure 3B, and Data S3). Cluster 7 had the highest average VAF in both GBM and SGS (GBM: 58.1%; SGS: 50.6%; Figure 3C and Data S3) and was inferred to be the founding clone, indicating that other subclones were derived from Cluster 7; this cluster contained 11 mutations. Cluster 0 and cluster 2 comprised mostly mutations having the lowest VAF. Cluster 0 was the largest cluster, containing 314 mutations, with mean VAF decreasing from 5.8% in GBM to nearly zero in SGS. Cluster 2 contained low‐frequency mutations, with mean VAF increasing from 1.0% in GBM to 3.0% in SGS. Cluster 1 had a low mean VAF of 1.0% in GBM and a moderate mean VAF of 15.0% in SGS, representing a subclonal population of tumor cells that significantly expanded during tumor evolution. Cluster 5 had a mean VAF of 21.0% in GBM and 13.2 in SGS, representing a subclonal population of tumor cells that had undergone clonal contraction during tumor relapse.

Notably, Cluster 7 contained a *NF1* mutation. The same mutation site, A690D/c.2069 C>A, was detected in both GBM and SGS, and the VAF of *NF1* was 50.0% in GBM and 51.1% in SGS (Figure 3D). *NF1* is a tumor‐suppressor gene; a mutation in this gene causes neurofibromatosis type 1. Various studies have identified somatic *NF1* mutations in non‐NF1‐associated cancers including GBM,^33^ and 10% of GBM tumors harbor an *NF1* somatic mutation.^34^ Considering that the VAF of *NF1* was nearly 50%, that the mutation was heterozygous, and that no CNVs were detected in the *NF1* gene region, we conclude that this *NF1* mutation must have been present in nearly all the tumor cells of primary GBM and SGS, indicating that the two components of SGS may originate from the same ancestral cancer cell.

Another important cancer‐driving gene in cluster 7 was *TP53*. Only one mutation site in *TP53* (V203 M/c.607 G>A) was observed in our study. The VAF of this *TP53* mutation increased from 26.7% in GBM to 50.8% in SGS (Figure 3D), indicating that the cellular prevalence of *TP53* mutation increased from being present in approximately half of the tumor cells to virtually all of the tumor cells. A single cell from the founding clone likely acquired this *TP53* mutation before the patient underwent anti‐GBM therapy, and only clones containing the *TP53* mutation survived after resection and radiotherapy. Consequently, the clone containing both *NF1* and *TP53* mutations became the founding clone for gliosarcoma.

### 3.4 Mutational signatures

**FIGURE 4:**
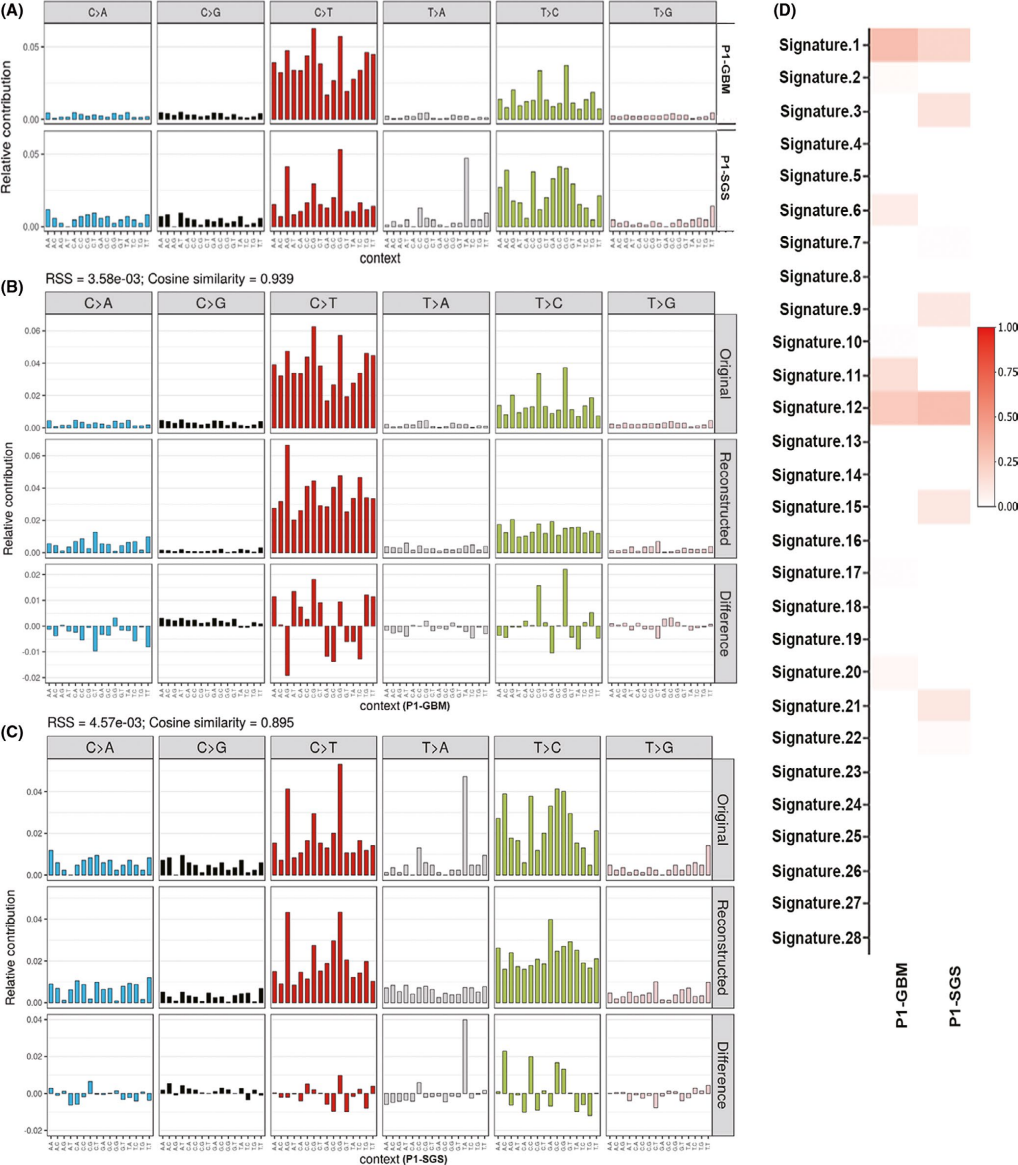
Mutation signatures in tumor tissues. A) Mutation prevalence and profiling of somatic mutations. All possible single nucleotide variants (SNVs) involving base substitution, and 5′and 3′adjacent nucleotides, are depicted. B, C: Reconstructed mutation signatures in glioblastoma multiforme (GBM) (B) and secondary gliosarcoma (SGS) (C). Vertical axis indicates the relative contribution of each mutation type. (D) Visualization of the contribution of COSMIC‐reported signatures. Relative contribution of each COSMIC signature is indicated by a corresponding key, as shown on the right

The overall frequency of SNVs identified in our WES data was 127.2 SNVs/Mb for GBM and 28.2 SNVs/Mb for SGS. SNV mutational signatures can provide genomic evidence for the etiology of tumor initiation and progression. Therefore, the mutational patterns of SNVs and small indels were used to evaluate whether the SGS examined in our present study could have resulted from exposure to therapeutic radiation. Radiation is known to cause DAN lesions *via* induction of reactive oxygen species (ROS). An increased proportion of C>A (G>T) mutations was observed in SGS compared with that in GBM (Figure 4A‐C). Additionally, 8‐oxoguanine is one of the most common ROS‐induced DNA‐base lesions. Misrepair of 8‐oxoguanine with adenine during DNA replication can generate C>A (G>T) transversion mutations.^35^ Thus, overpresented ROS‐associated C>A (G>T) substitutions in SGS may be an indirect consequence of therapeutic radiation. Surprisingly, signature 18, which had previously been reported by studies on ROS‐induced DNA lesions, was not found in SGS (Figure 4D). Compared with that of GBM, a higher contribution of COSMIC signature 3, 9, 12, 15, and 21 was observed in SGS (Figure 4D). Although the relationship between these signatures and radiation exposure is still unclear, most of these signatures are associated with defects in DNA repair.^36^ Interestingly, signature 11 was not found in the SGS examined in our study (Figure 4D). Because signature 11 is characterized predominantly by C>T (G>A) mutations and is associated with exposure to alkylating agents,^37^ the DNA damage observed in this case of SGS was likely the result of radiotherapy rather than mutagenic temozolomide therapy.

### 3.5 Mutation incidences of *NF1* and *TP53* in GBM patients from TCGA database

**FIGURE 5:**
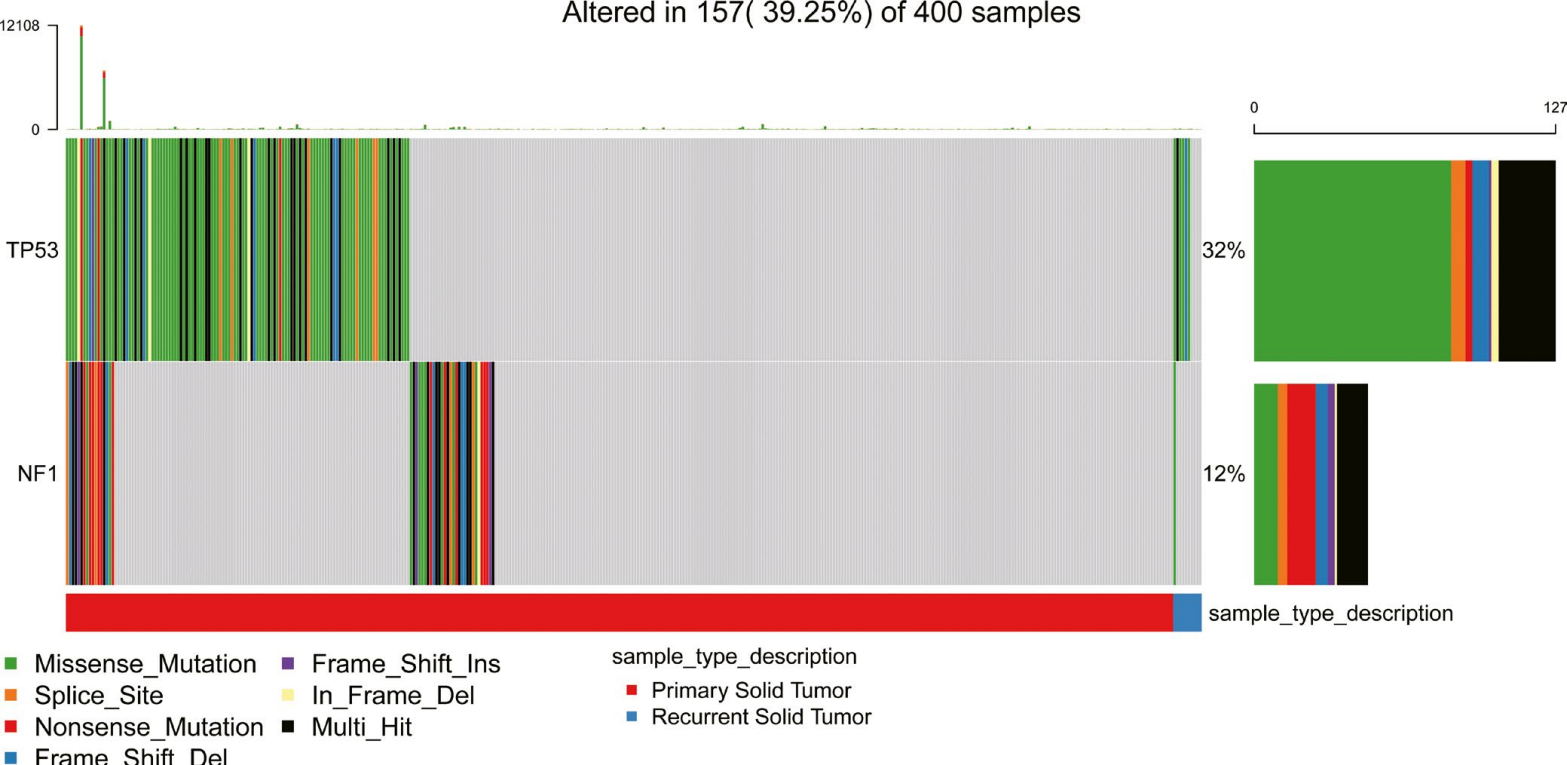
Somatic alterations in *TP53* and *NF1* in glioblastoma multiforme (GBM) patients from TCGA database. There are 390 primary and 10 recurrent GBM samples. In primary GBM patients, the somatic alterations of *TP53* and *NF1* occurred in 121 and 47 samples, respectively. In recurrent GBM patients, the alterations of *TP53* and *NF1* occurred in 6 and 1 samples, respectively

To determine whether *NF1* and *TP53* mutations commonly occur in primary GBM, we searched the data of these mutations from TCGA, CGGA, and GEO databases. Finally, the relevant data were screened out only from TCGA database, including 390 primary GBM samples (without chemoradiotherapy before surgical resection) and 10 recurrent GBM (occur after radiochemotherapy). The overall incidences of *TP53* and *NF1* alterations in primary GBM patients are 31% (121/390) and 12% (47/390), respectively (Figure 5).

We also attempted to investigate whether radiotherapy increased the incidence of *NF1* and *TP53* mutations by comparing the mutations rates between the paired primary (without radiotherapy) and recurrent (occur after radiotherapy) GBM cases from TCGA. However, there were only nine paired cases in the database, and most of them received both radiotherapy and chemotherapy (data not shown). Therefore, we did not perform any further analysis of the mutation incidence after radiotherapy.

## 4 DISCUSSION

Gliosarcoma, a bimorphic tumor, contains both gliomatous and sarcomatous components. The glial regions in gliosarcoma are highly similar to those observed in GBM, while the sarcomatous region consists of neoplastic mesenchymal cells with associated reticulin formation. Cases of SGS occurring after initially diagnosed GBM are exceedingly rare, with only a limited number of such described in the literature. The histogenesis and origin of SGS remain controversial. Feigin and Gross were the first to detail gliosarcoma and proposed that the sarcomatous elements arose from neoplastic transformation of blood vessels induced by the malignant glial cells.^38^ However, this hypothesis is hindered by inconsistent expression of vascular markers in the sarcomatous component. Some studies have proposed that the sarcomatous component is derived from pluripotent mesenchymal cells of perivascular adventitia, fibroblasts, or even histocytes.^8^, ^9^, ^10^, ^11^ However, genetic studies have identified a similar genetic profile in both components,^12^, ^13^, ^14^, ^15^, ^16^ strongly indicating that gliosarcoma has a monoclonal origin. In the case described herein, a cluster of mutations was shared in both primary GBM and SGS, including a founding mutation in the two tumor suppressor genes *NF1* and *TP53*. Therefore, our results also indicate that gliosarcomas have a monoclonal origin.

Most patients with SGS were previously managed with radiotherapy for the original GBM.^19^, ^32^, ^39^ Gliosarcoma can also occur after radiation therapy in patients with other tumor phenotypes (e.g., leukemia, meningioma, and low‐grade glioma), which is termed radiation‐induced gliosarcoma. Some cases of radiation‐induced gliosarcoma develop within the radiation field but at a location separate from that of the primary tumor.^19^ These observations indicate a causal association between therapeutic radiation and induction of sarcomatous components. Several anticancer drugs, including alkylating agents, are also mutagenic. Because the patient described in our present study was administered both radiotherapy and treatment with temozolomide, we examined the mutational signatures of tumor tissues obtained from this patient in order to investigate the possible etiology of tumor evolution from GBM to SGS. Our results indicate that the prevalence of C>A (G>T) transversions was higher in SGS than in GBM. This mutational alteration is not in accord with the pattern induced by temozolomide, which is characterized by a predominance of C>T (G>A) mutations.^37^ Consistently, the contribution of signature 11 in SGS was scant; this signature is associated with exposure to alkylating agents. The C>A (G>T) transitions in SGS can be explained by ROS‐induced mutagenic DNA lesions following radiotherapy. Our results indicate that therapeutic radiation was a significant contributor to the somatic mutations observed in this SGS tumor.

The clone containing mutations in *NF1* and *TP53* may have been the founding clone in this case. The *TP53* gene, known as the guardian of the genome, is critical in detecting DNA damage and preventing damaged cells from passing the damaged DNA to their daughter cells. Our analysis of the genomic data from TCGA showed that the overall incidences of *TP53* mutations were 31% (121/390). Genetic studies have showed that gliosarcoma has a higher frequency of *TP53* mutation than primary GBM.^14^, ^32^ Consistently, we observed a higher prevalence of *TP53* mutations (V203M) in SGS compared with that in primary GBM. A recent study demonstrated that temozolomide can induce two *TP53* missense mutations (R110C/c.328 C>T and R175H/c.524 G>A) in glioma spheres derived from primary GBM, and that these mutations may facilitate epithelial‐to‐mesenchymal transition.^40^ These findings indicate that *TP53* mutations likely participate in driving the development of GBM to SGS. Two recent studies, examining molecular and genetic profiles in a gliosarcoma patient with multiple recurrences and an extracranial metastasis, showed that several somatic mutations that are key in primary gliosarcoma, such as a *TP53* mutation, were shared in recurrent and metastatic tumors.^41^, ^42^ Our analysis demonstrated that the overall incidences of *NF1* mutations were 12% (47/390) in primary GBM patients from TCGA cohort. The *NF1* mutation in gliosarcoma patients has also been reported in two studies, with the frequency of 21% (3/14)^32^ and 30% (3/10),^43^ respectively. Due to the small number of patients in the two reports, further studies are needed to reveal the overall incidence of *NF1* mutations in gliosarcoma. Tumor stem cells theory is an alternative explanation for tumor development and progression. Although our study indicated that the clone possessing mutations in *NF1* and *TP53* was the founding clone in this case, whether the clone originates from glioma stem cells or other progenitor cells is unknown. To investigate whether the glioma stem cells harbor *NF1* and *TP53* mutations, we have searched the mutation profile of glioma stem cells from two databases, including COSMIC Cell Lines Project (https://cancer.sanger.ac.uk/cell_lines/) and GSE23806 (download from GEO website, https://www.ncbi.nlm.nih.gov/geo/).^44^.However, we did not find the relevant information in the two databases.

Our results indicate that mutation rate decreased significantly in SGS (3.5 per Mb) as compared with that in GBM (7.2 per Mb). Interestingly, our GBM samples exhibited a high burden of somatic mutations and low CNVs, whereas our SGS samples showed a low burden of somatic mutations and high CNVs. The inverse relationship between these two genomic aberrations may indicate the presence of a compensatory mechanism in tumor evolution.^45^ When mutation load is high, the decline in CNVs is driven by cellular autonomous mechanisms and immune response to neoantigens. When mutation load decreases following chemoradiotherapy, CNVs increase because of suppression of immune surveillance in the tumor microenvironment. Further studies are necessary to delineate the dynamic correlations between these genomic aberrations.

In conclusion, herein, we described the first genome‐wide deep sequencing of paired primary GBM and SGS samples obtained from the same patient. Our results provide genomic evidence for the monoclonal origin of the two components of SGS and for the relationship between therapeutic radiation and SGS pathogenesis. Therapy‐driven tumor evolution is a major impediment in the management of GBM. Improving our understanding of the molecular and genetic mechanisms driving therapy‐driven tumor evolution will facilitate the development of more effective therapeutic strategies. Due to the rarity of SGS, only one patient with SGS was described in this study. Future studies enrolling more patients will help reveal the mechanisms involved in the transformation of GBM into SGS.

## CONFLICT OF INTERESTS

The authors declare that this study was conducted in the absence of any commercial or financial relationships that could be construed as a potential conflict of interest.

## Supporting information

Data S1

Data S2

Data S3

## Data Availability

The sequencing data were deposited into the Sequence Read Archive database under accession number PRJNA720573. These data will be made available to the public after publication of this manuscript.
